# Inducing bursicon expression using 20-hydroxyecdysone (20E) increased immune response in *Macrobrachium rosenbergii* against *Aeromonas hydrophila*

**DOI:** 10.1242/bio.061773

**Published:** 2025-07-16

**Authors:** Arnon Pudgerd, Sukanya Saedan, Thanapong Kruangkum, Kallaya Sritunyalucksana, Sirilak Sanpa, Sudthiporn Somnet, Rapeepun Vanichviriyakit, Charoonroj Chotwiwatthanakun

**Affiliations:** ^1^ Division of Anatomy, School of Medical Science, University of Phayao, Muang, Phayao 56000, Thailand; ^2^ Unit of Excellence in Plant and Aquaculture, University of Phayao, Muang, Phayao 56000, Thailand; ^3^ Center of Excellence for Shrimp Molecular Biology and Biotechnology (Centex Shrimp), Faculty of Science, Mahidol University, Rama VI Road, Bangkok 10400, Thailand; ^4^ Department of Anatomy, Faculty of Science, Mahidol University, Rama VI Road, Bangkok 10400, Thailand; ^5^ Aquatic Animal Health Research Team (AQHT), Integrative Aquaculture Biotechnology Research Group, National Center for Genetic Engineering and Biotechnology (BIOTEC), National Science and Technology Development Agency (NSTDA), Bangkok 10400, Thailand; ^6^ Microbiology Program, School of Medical Science, University of Phayao, Muang, Phayao 56000, Thailand; ^7^ Nakhonsawan Campus, Mahidol University, Nakhonsawan 60130, Thailand

**Keywords:** 20-hydroxyecdysone, *Macrobrachium rosenbergii*, Bursicon neurohormone, Immune response, *Aeromonas hydrophila*

## Abstract

A bursicon (burs) is a neurohormone that regulates cuticle tanning after molting, as well as the immune response, in insects and crustaceans. This study investigated the relationship between ecdysteroids, burs expression and immune regulation following 20-hydroxyecdysone (20E) injection in *Macrobrachium rosenbergii*. Burs subunits (burs α and burs β) were highly expressed in the thoracic ganglia during the late pre-molt stage (D_3_). Significant upregulation of the *burs α* and *burs β*, *anti-lipopolysaccharide factor* (*ALF*) and *crustacean hematopoietic factor* (*CHF*) genes accompanied an increase in the hemocyte concentration. The efficacy of immune enhancement for disease protection from 20E injection showed that 20E treatment upregulated *burs* genes in thoracic and abdominal ganglia. *ALF* and *CHF* expression and phenol oxidase activity were significantly increased. The hemocyte proliferation percentage in hematopoietic tissue increased 12 h post-infection, while circulating hemocytes increased significantly within the first 24 h. Administering 20E decreased mortality in *Aeromonas hydrophila*-challenged prawns. This study demonstrated that the ecdysteroid 20E stimulated burs expression and improved the immune response to bacterial challenge, suggesting that this hormone plays a role in regulating the immune system during ecdysis.

## Teaser

**Summary:** Ecdysteroid stimulated burs expression and improved the immune response against bacterial challenges.

## INTRODUCTION

*Macrobrachium rosenbergii* is an economically important species for tropical and subtropical regions, including Thailand (Jariyapong et al., 2019, 2020). Major losses of a stocking prawn have arisen from bacterial and viral infectious diseases (Cheng et al., 2003). Although ecdysis is an essential biological process that establishes growth and development patterns in crustaceans (Hyde et al., 2019), it also alters immune responses and susceptibility to microbial infections (Cheng et al., 2003). Thus, an essential understanding of the molting mechanisms underlying immune responses can serve as foundational knowledge for improving the quality of *M. rosenbergii* culture.

In arthropods, ecdysis is a fundamental biological process that underlies the growth, development, and regeneration of the exoskeleton. Ecdysone (20-hydroxyecdysone, 20E) is a central endocrine regulator of molting and metamorphosis. It binds to receptors on the nuclear membrane of the target cell, controlling the expression of numerous genes (Hyde et al., 2019). Sloughing of the exocuticle during molting increases the risk of microbial invasion due to the loss of the physical barrier that it provides. Immune regulation is necessary to protect against pathogen infection during this critical period. In *Drosophila*, ecdysone activates hemocytes through their receptor (ecdysteroid receptor, EcR) on the cell surface during metamorphosis (Regan et al., 2013; Sampson et al., 2013). This signaling is necessary for hemocyte motility (Sampson et al., 2013) and hemocyte activation to combat bacterial infection (Regan et al., 2013).

The negative effects on stress responses and immunity modulation are evident in crustaceans during molting. At the pre- and post-molt stages, *Penaeus semisulcatus* are more sensitive to ammonia toxicity and less resistant to environmental stress (Wajsbrot et al., 1990). *M. rosenbergii* is more susceptible to *Lactococcus garvieae* infection during molting (Cheng et al., 2003). An adverse effect during molting has been demonstrated, with a decrease in the expression of p38 mitogen-activated protein kinase (MAPK), involved in regulating innate immunity (He et al., 2013). It was initially shown that immune factor suppression occurs during molting; however, the immune-regulatory and antipathogenic functions of ecdysteroids remain a mystery. Injection of 20E has bimodal effects on the immune system. A study on *Penaeus vannamei* demonstrated that ecdysteroid reduced the total hemocyte count (THC), phenol oxidase activity (PO), and O_2_^−^, while increasing the activity of certain immune-related genes, e.g. superoxide dismutase (SOD), glutamate pyruvate transaminase (GPT), and glutamate oxaloacetate transaminase (GOT) (Wu et al., 2016), but no such information is available for *M. rosenbergii*. The ecdysone level and ecdysone receptor (EcR) expression increase following lipopolysaccharide (LPS) injection, indicating the importance of ecdysone and its receptor in immune activation in *Eriocheir sinensis* (Huang et al., 2020). These studies suggest that ecdysteroids may have dual immune-regulatory effects.

During molting, a positive feedback loop involving neuropeptides such as eclosion hormone and ecdysis-triggering hormone causes a significant release of peptides into the hemolymph. This activates the neuronal network that regulates ecdysis and post-ecdysis behavioral sequences, resulting in the production of cyclic GMP (cGMP), crustacean cardioactive peptide (CCAP), burs, and other neuropeptides (Lenaerts et al., 2017). Burs is a neuropeptide hormone that plays a role in the tanning process and is involved in post-molting development. It consists of two cystine knot subunits: α (burs α) and β (burs β). A burs heterodimer regulates cuticle tanning, integumentary structure development, and maturation and is also involved in the immune response mechanism (An et al., 2008; Li et al., 2019). Previous studies in *Drosophila* demonstrated its function in the genetic basis of immunity (An et al., 2008), immune regulation during stress in *M. rosenbergii* (Jariyapong et al., 2020), and infection in *Procambarus clarkii* (Zhang et al., 2020). Burs homodimers mediate signal transduction via Relish, a nuclear factor-kB (NF-kB) transcription factor homolog, to induce the expression of antimicrobial peptide genes such as anti-lipopolysaccharide factor (ALF) and crustin (An et al., 2012; Zhang et al., 2020). Additionally, during infection, *M. rosenbergii* increases the expression of crustacean hematopoietic tissue (CHF), which plays a role in hemocyte homeostasis. CHF mRNA is expressed in both viral and bacterial infections (Jariyapong et al., 2019; Pudgerd et al., 2021). Although the levels of ecdysteroids are related to the induction of a cascade of multiple neuropeptides (Malhotra and Basu, 2023), there has not yet been a study showing that ecdysteroid hormones can stimulate the expression of bursicon. Additionally, the relationship between burs and ecdysteroids in terms of immune regulation has never been elucidated.

This study characterized the expression of burs genes across different molting stages in *M. rosenbergii*. To clarify the hormonal interplay between burs and ecdysteroids, a 20E injection was administered. Immune potency and the response to 20E injection were assessed using immune parameters such as hematopoietic cell proliferation and immune-related gene expression. Additionally, the role of immune potentiation in disease protection following 20E injection was examined in relation to *A. hydrophila* infection. The outcomes of the study will explain how molting-related hormones regulate the innate immune response to pathogens at the molecular level.

## RESULTS

### The expression of *burs α* and *burs β* is associated with the pre-molting period in *M. rosenbergii*

**Fig. 1.**
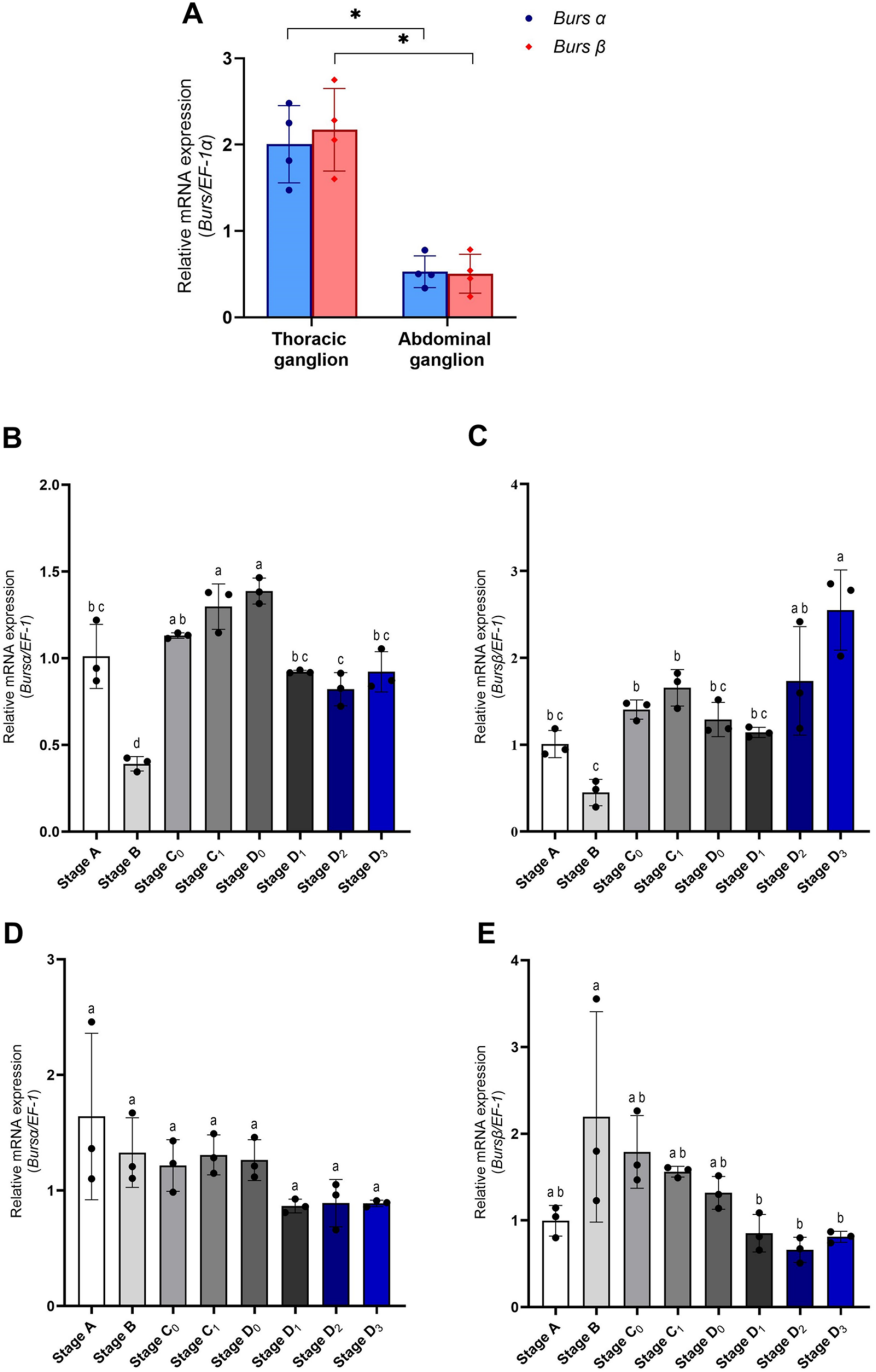
**Quantitative RT-PCR determination of *burs α* and *burs β* in the thoracic and abdominal ganglia.** (A) Transcription level of *burs α* and *burs β* in the thoracic and abdominal ganglia (*n*=4). (B-E) Expression profile of *burs α* and *burs β* in the thoracic and abdominal ganglia during the molt cycle. (B) Expression of *burs α* and (C) expression of *burs β* in the thoracic ganglia. (D) Expression of *burs α* gene and (E) expression of *burs β* in the abdominal ganglia (*n*=3). Asterisks indicate statistically significant differences *P<0.05. Different letters indicate significant differences between the molt stages (*P<*0.05).

The expression profiles of *burs α* and *burs β* were previously observed in the thoracic and abdominal ganglia, but their levels were unknown. In this study, the expression of *burs α* and *burs β* in the thoracic and abdominal ganglia was evaluated quantitatively, and the results are shown in Fig. 1A. The expression of both *burs* forms was significantly higher in the thoracic ganglia than in the abdominal ganglia (*P*<0.05). However, the expression levels of *burs α* and *burs β* were not different within the tissue.

*Burs* expression was investigated quantitatively across molting stages, and the results are shown in Fig. 1B-E. The expression level of *burs α* in the thoracic ganglia decreased in stage B after post-molting stage A, gradually increased in stages C_0_-D_0_, and then gradually decreased again in stage D_1_. The relative expression level of *burs α* was very low in stage D_2_ and gradually increased in stages D_3_-A (Fig. 1B). The expression of *burs β* in the thoracic ganglion was similar to that of *burs α* during stages A-D_1_, but much higher during stages D_2_-D_3_ (Fig. 1C). In the abdominal ganglion, the relative expression of *burs α* gradually decreased during the molting stage, starting with the highest expression at stage A (Fig. 1D). By contrast, the relative expression of *burs β* decreased in stage A and was highly expressed in stage B. Then, the expression of *burs β* gradually decreased from stage C_0_ to D_3_ (Fig. 1E).

### Immune parameters increase during the pre-molting stage

**Fig. 2.**
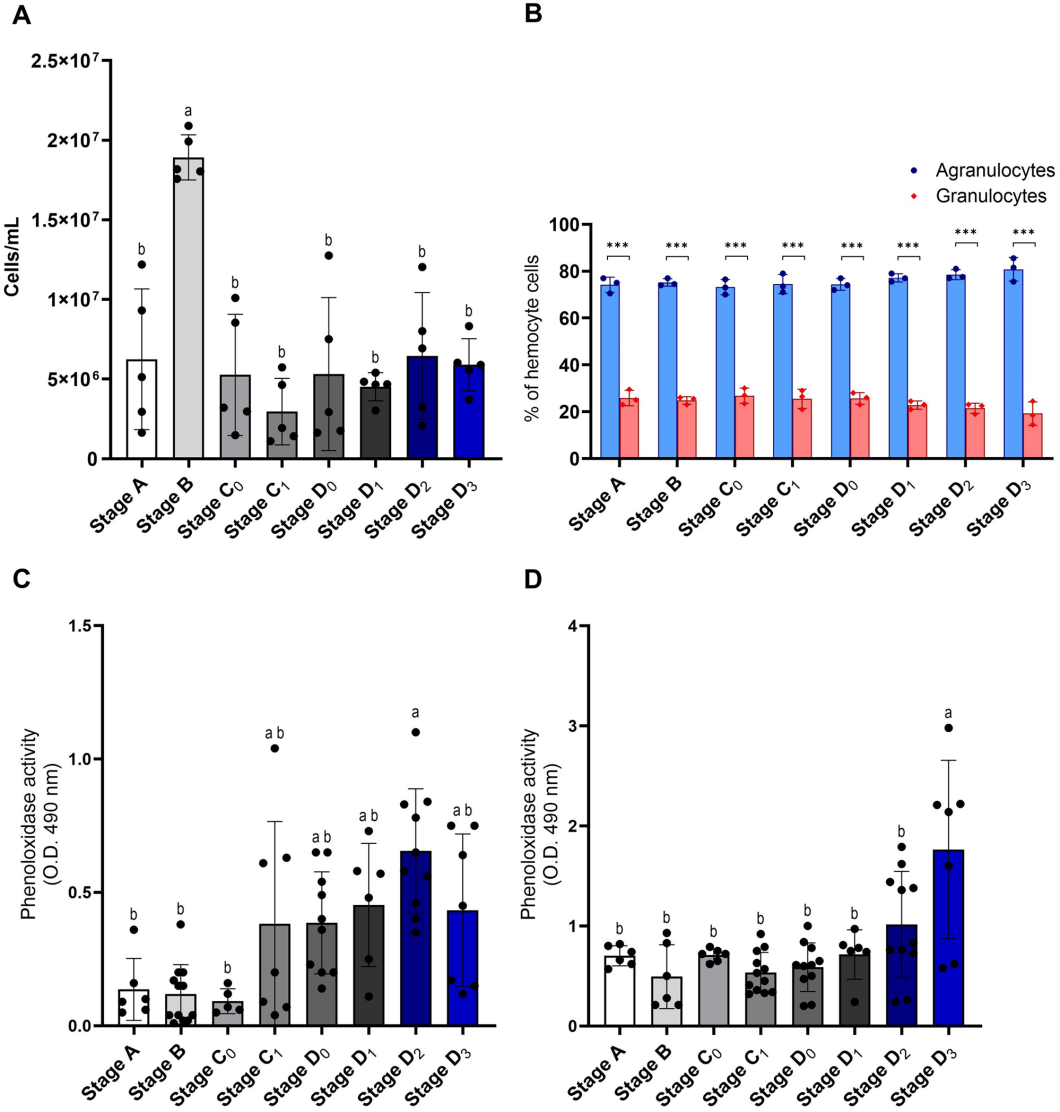
**Hemocyte circulation and phenoloxidase activity during the molt cycle.** (A) The change of the hemocyte concentration in all stages of the molt cycle (*n*=5). (B) The ratio of agranulocyte and granulocyte density in all stages of the molt cycle (*n*=3). (C) The PO activity of hemocytes and (D) the PO activity of hemolymph of *M. rosenbergii* show a gradual increase in the premolt stage (*n*=5-12). Different letters indicate significant differences between the molt stages (*P<*0.05). Asterisks indicate statistically significant differences ***P<0.001.

During the molting cycle, there was a notable increase in hemocyte circulation at stage B compared to stages A, C_0_, C_1_, D_0_, D_1_, D_2_, and D_3_ (*P*<0.05). Additionally, hemocyte circulation decreased in other molting stages, but the difference was not significant (C_0_-D_3_ and A) (Fig. 2A). For DHC, the number of agranulocytes was significantly higher than that of granulocytes (*P<*0.001) at all stages. However, the numbers of both agranulocytes and granulocytes did not differ during the molt cycle (Fig. 2B). PO activity in hemocytes significantly increased at stage D_2_ compared to stages A, B, and C_0_ (*P*<0.05). Although the other stages showed no significant differences, the activity of PO gradually increased during the pre-molt stage (D_0_-D_2_) compared to the post-molt stage (stages A-C_0_) (Fig. 2C). This PO activity pattern was also observed in the hemolymph, with the pre-molt stage (D_3_) showing a significant increase compared to stages A-D_2_ (*P*<0.05) (Fig. 2D).

### A higher level of *burs α* and *burs β* expression, along with increased immune parameters, is observed following 20E induction

**Fig. 3.**
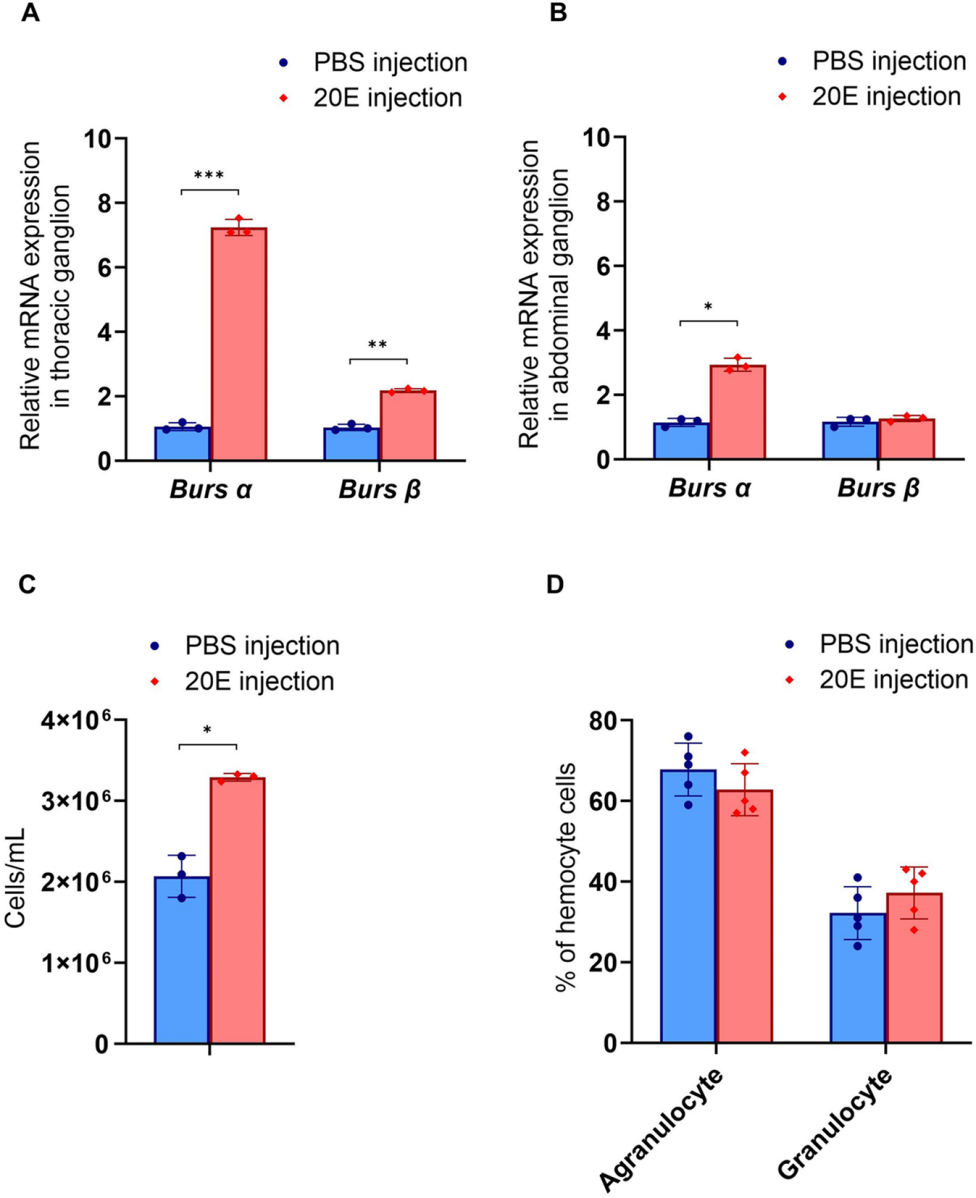
**Expression of *burs α* and *burs β* and determination of hemocyte circulation following 20E injection.** Three hours after 20E injection, the expression of *burs α* and *burs β* was determined by qRT-PCR in the thoracic ganglia (A) and abdominal ganglia (B) (*n*=3). The hemocyte density (C) and the ratio of agranulocyte to granulocyte density (D) change after 20E injection (*n*=5). Asterisks indicate statistically significant differences (*P<0.05, **P<0.01, ***P<0.001).

**Fig. 4.**
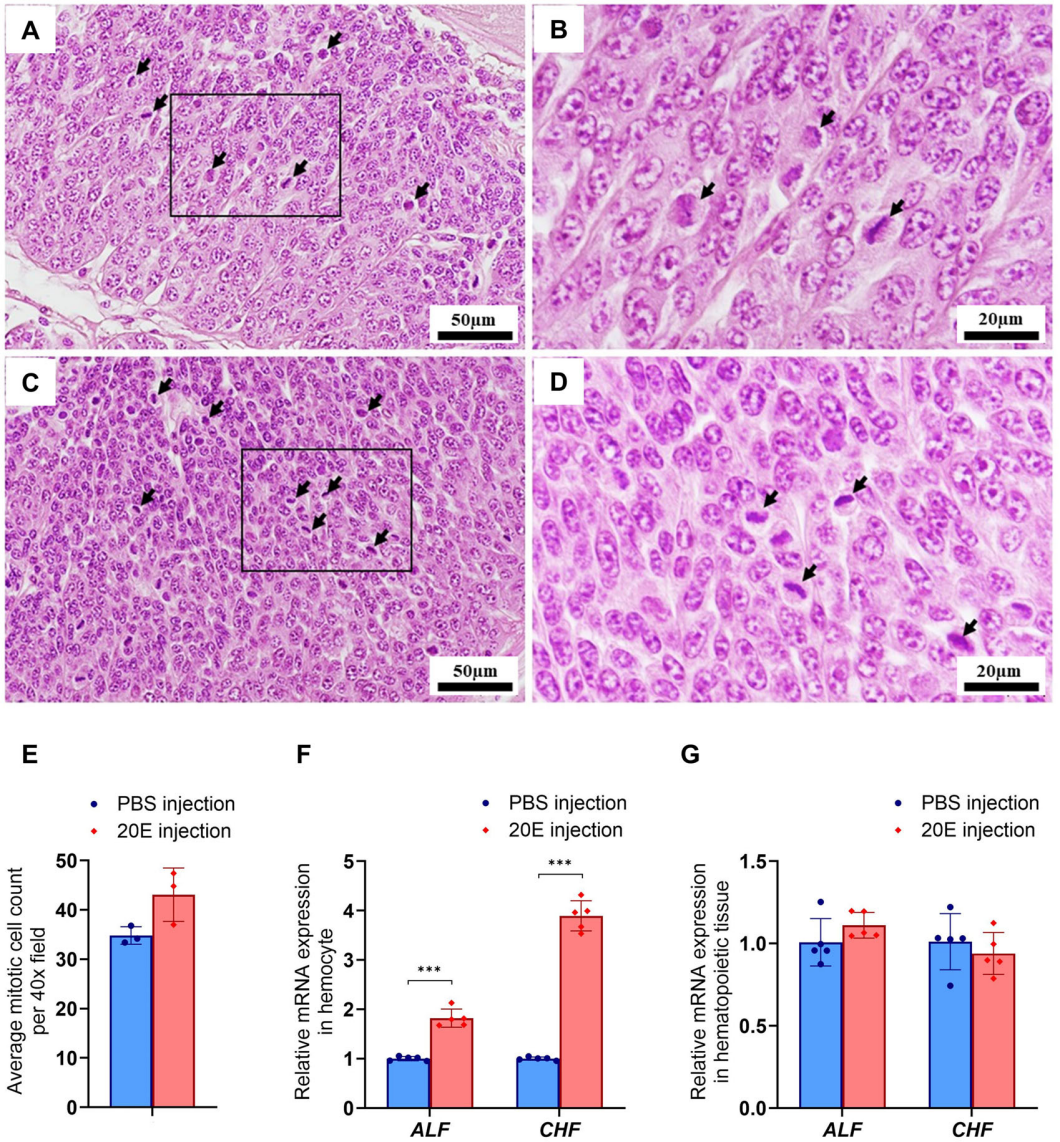
**Hematopoietic cell proliferation and expression of immune-related genes following 20E injection.** Histological section of hematopoietic tissue showing mitotic cells after injection with PBS (A,B) and 20E (C,D). Black arrows indicate the mitotic cells. (E) Histogram showing a higher average number of mitotic cells in hematopoietic tissue following 20E compared to PBS injection. Histogram demonstrating an increase in *ALF* and *CHF* mRNA expression in hemocytes (F) following 20E injection, while the expression of *ALF* and *CHF* mRNA was consistent in hematopoietic tissue (G) (*n*=5). Asterisks indicate statistically significant differences (***P<0.001).

After 20E injection, mRNA expression levels of *burs α* and *burs β* were significantly increased in the thoracic ganglion compared to the control group (*P*<0.001, *P*<0.01, respectively) (Fig. 3A). There was also a significant increase in the relative expression of *burs α* in the abdominal ganglion (*P*<0.05) (Fig. 3B). Furthermore, the prawn that received 20E also demonstrated a significant increase in hemocyte circulation (*P*<0.05) (Fig. 3C). However, there was no difference between the numbers of agranulocytes and granulocytes (Fig. 3D). Increased hemocyte circulation was related to hematopoietic tissue cell proliferation, as indicated by mitotic figures (arrow) (Fig. 4A-E). More mitotic cells were observed in the 20E injection group than in the control group, although the difference was not significant (Fig. 4E). Additionally, there was a significant increase in the relative expression of *ALF* (*P<*0.001) and *CHF* (*P<*0.001) of hemocytes in the 20E injection group compared to the control group (Fig. 4F). However, the expression of these genes did not differ in the hematopoietic tissue between the two groups (Fig. 4G). The expression of immune-related genes and *burs* in the same neuroendocrine tissues upon 20E injection suggested that 20E modulates the immune response through *burs α* and *burs β*.

### Evaluating innate immune potency against *A. hydrophila* infection in *M. rosenbergii* using 20E treatment

#### Treatment with 20E stimulated *burs α* and *burs β* expression

**Fig. 5.**
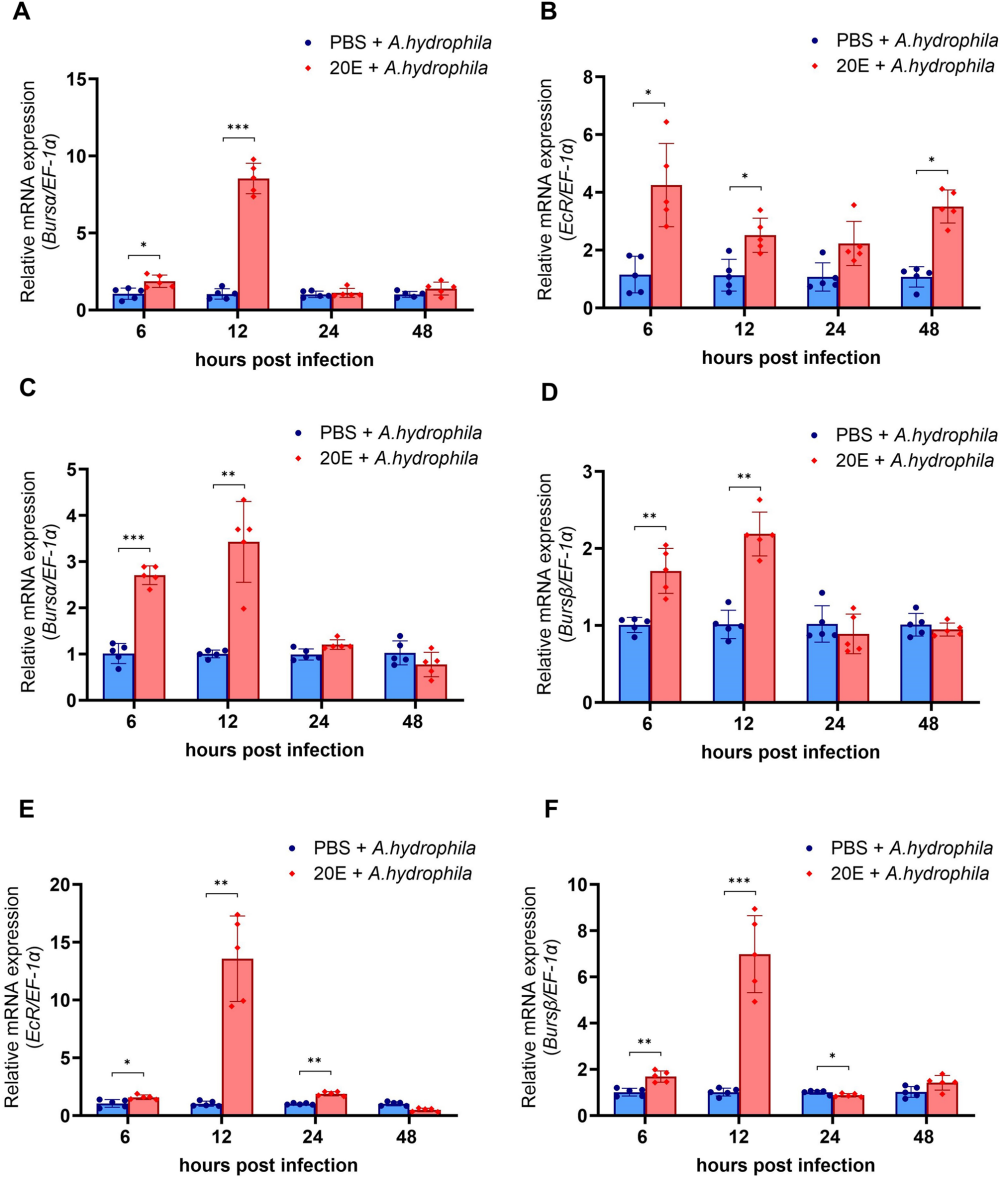
**Expression of *EcR*, *burs α,* and *burs β* in prawn infected with *A. hydrophila* and 20E injection.** The expression of *EcR*, *burs α*, and *burs β* in the thoracic ganglia (A,C, and D) and the abdominal ganglia (B,E, and F) was analyzed using qRT-PCR at 6, 12, 24, and 48 h post-injection during PBS and 20E treatment in *A. hydrophila* infection (*n*=5). The asterisks indicate significant differences between the PBS and 20E treatment groups at each time point (*P<0.05, **P<0.01 and ***P<0.001).

Following treatment with PBS and 20E and subsequent *A. hydrophila* infection, *EcR* expression increased in the thoracic ganglion of *M. rosenbergii* (Fig. 5A) at 6 h (*P*<0.05), 12 h (*P*<0.01), and 24 h (*P*<0.05). Moreover, *EcR* upregulation was detected in the abdominal ganglion at all time points, with a significant increase at 6, 12, and 48 h (*P*<0.05) post-infection (Fig. 5B). This upregulation of *EcR* was also observed alongside that of *burs α* and *burs β* in the thoracic (Fig. 5C,D) and abdominal ganglia (Fig. 5E,F). The expression of *burs α* was significantly upregulated in the thoracic ganglion 6 h (*P*<0.001) and 12 h (*P*<0.01) post-infection (Fig. 5C) and in the abdominal ganglion 6 h (*P*<0.05) and 12 h (*P*<0.001) post-infection (Fig. 5E). *burs β* gene expression was significantly increased in both the thoracic (Fig. 5D) and abdominal ganglia (Fig. 5F) at 6 and 12 h post-infection. There was no significant difference in *burs β* gene expression in the thoracic ganglion between the PBS and 20E treatment groups at 24 and 48 h post-infection (Fig. 5D). However, *burs β* expression was gradually downregulated 24 h post-infection (*P*<0.05) and returned to being upregulated at 48 h post-infection in the abdominal ganglion (Fig. 5F). These results suggested that 20E can activate the expression of the *burs α* and *burs β* genes to promote the expression of immune parameters.

#### Hematopoietic cell proliferation increased with 20E treatment

**Fig. 6.**
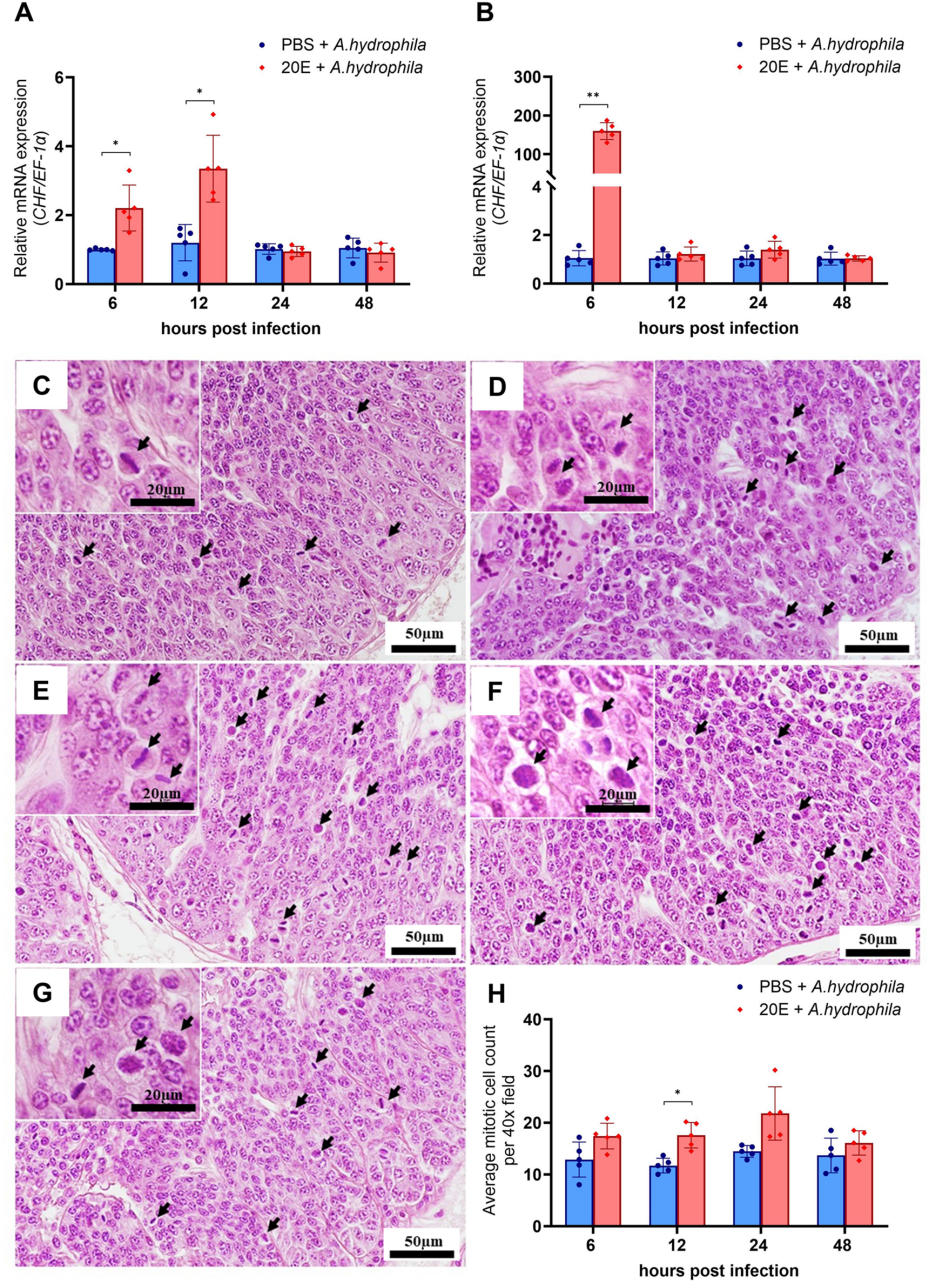
**Expression of *CHF* and hematopoietic cell proliferation in prawn infected with *A. hydrophila* and 20E injection.** The *CHF* mRNA expression in the hemocytes (A) is upregulated 6-12 h post-infection, while in the hematopoietic tissue (B), it is upregulated at 6 h post-infection. Histological section of hematopoietic tissue showing reduced hematopoietic cell proliferation in the PBS treatment group at various time points following bacterial infection, as represented by the hematopoietic tissue at 6 h post-infection (C). In the 20E treatment groups, hematopoietic tissue exhibits a high level of proliferation at 6 h (D), 12 h (E), 24 h (F), and 48 h (G) post-infection. Black arrows indicate cells undergoing mitotic cell division. The average number of mitotic cells in hematopoietic tissue is counted (H). The asterisk indicates significant differences among treatments at each time point (*n*=5, *P<0.05 and ***P<0.001).

During infection, *CHF* expression was significantly upregulated in hemocytes between 6 and 12 h post-infection (*P*<0.05) (Fig. 6A) and highly upregulated in hematopoietic tissue at 6 h post-infection (*P*<0.001) (Fig. 6B). Histological analysis revealed that hematopoietic cell proliferation was reduced in the PBS treatment group (Fig. 6C), whereas the 20E treatment group exhibited increased proliferation (Fig. 6D-G), with black arrows in the figures indicating dividing cells. Across all time points, the average number of mitotic cells in the hematopoietic tissue of *M. rosenbergii* treated with 20E was higher, but the increase was statistically significant only at 12 h post-infection (Fig. 6H).

#### Treatment with 20E increased the survival rate and immune-related gene expression

**Fig. 7.**
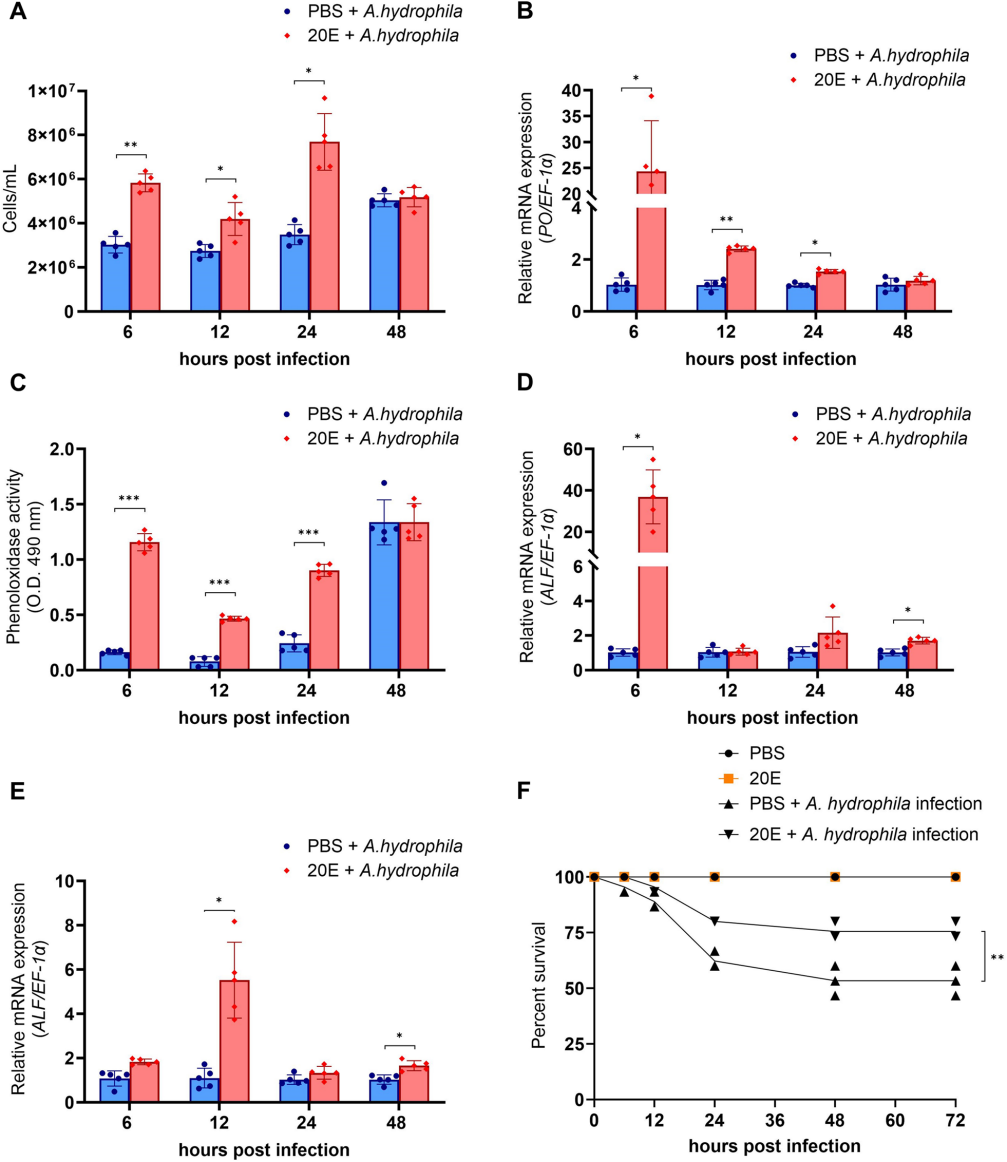
**Evaluation of immune parameters and survival rate of *A. hydrophila* challenged prawn following 20E injection.** (A) Histogram demonstrating a significant increase in total hemocyte count in the hemolymph of prawns treated with 20E during *A. hydrophila* infection between 6 and 24 h (*n*=5). In the 20E-treated group, the expression level of *phenol oxidase* (PO) in hemocytes was upregulated between 6 and 24 h post-infection (*n*=4) (B), and PO activity in the hemolymph significantly increased between 6 and 24 h (*n*=5) (C). Moreover, the expression level of *ALF* in the hemocytes (*n*=4) (D) increased at 6 and 48 h, while it was elevated in the hematopoietic tissue (*n*=4) (E) between 6 and 12 h and at 48 h. Cumulative survival against *A. hydrophila* infection of *M. rosenbergii* indicated a significant increase in the 20E treatment group compared to the PBS treatment group (*n*=4) (F). Asterisks indicate significant differences among treatments at each time point (*P<0.05, **P<0.01, and ***P<0.001).

In *A. hydrophila* infection, the analysis of immune parameters showed that the 20E treatment group significantly increased hemocyte circulation between 6 and 24 h after infection at *P*<0.01, *P<*0.05, and *P<*0.05, respectively, compared to the PBS treatment group (Fig. 7A). The mRNA expression of *proPO* in hemocytes was significantly increased in the 20E treatment group between 6 and 24 h after infection (*P<*0.05, *P<*0.01, and *P<*0.05, respectively) (Fig. 7B). This gene expression pattern corresponded to that of PO activity in the hemolymph (Fig. 7C). Furthermore, *ALF* mRNA expression significantly increased after 6 h of 20E treatment (*P<*0.001). However, there was no significant difference in gene expression between the PBS and 20E treatment groups between 12 and 24 h post-infection, and it gradually increased again after 48 h (*P<*0.05) (Fig. 7D). The pattern of *ALF* expression in hematopoietic cells was upregulated at 6 h and significantly increased at 12 h post-infection (*P*<0.05), and there was no difference between PBS and 20E treatment at 24 h. However, this gene was again upregulated at 48 h post-infection (*P*<0.05) (Fig. 7E). This study also found that increasing these immune parameters enhanced the survival rate of *M. rosenbergii*. Between 0 and 48 h, the survival rate of infected *M. rosenbergii* significantly increased in the 20E-treated group to 76.00%, compared with 53.33% in the PBS-treated group. When compared with the survival rate of the uninfected group (100%; *n*=15), that of infected prawns receiving 20E decreased by 24.00% (*n*=3.6), while the survival rate of the infected group treated with PBS decreased by 46.67% (*n*=7) (*P*<0.01) (Fig. 7F). There was no significant difference in survival rates between the uninfected groups treated with PBS and 20E.

## DISCUSSION

Burs hormone, a neuropeptide hormone belonging to the cystine-knot protein family, consists of burs α and burs β. The burs heterodimer signals through the G protein-coupled receptor DLGR2 to mediate insect cuticle tanning and other developmental processes (Zhang et al., 2017). Burs are found in the ventral nervous system of *D. melanogaster* and specific neurons of *Teleogryllus commodus* and *Periplaneta americana* (Luo et al., 2005), as well as in the abdominal nerve cord of *Homarus americanus* (Kostron et al., 1995), and both the thoracic and abdominal ganglia of *M. rosenbergii* (Suwansa-Ard et al., 2015). After the burs bind to their receptor, cAMP is produced, activating cAMP-dependent protein kinase to phosphorylate tyrosine hydroxylase, which converts tyrosine to 3,4-dihydroxyphenylalanine and facilitates cuticle tanning (Luo et al., 2005; Zhang et al., 2017). In this study, the expression of burs subunits was similar to that in *Aedes aegypti* (Zhang et al., 2017) and *Anopheles gambiae* (Honegger et al., 2011). The homodimer form of burs activates the immune system (Li et al., 2019). Eighty-seven genes downstream of *burs* are activated, including seven genes involved in the immune system (An et al., 2008). In this study, the thoracic ganglia exhibited higher expression levels of *burs α* and *burs β* than the abdominal ganglia, suggesting that the primary site of burs synthesis is in the thoracic ganglia of *M. rosenbergii*. However, the expression of *burs α* and *burs β* varied across tissues. In *Neocaridina heteropoda*, both subunits were primarily synthesized in the abdominal ganglia rather than other organs (Li et al., 2019). This differential expression may be influenced by organ size and/or the number of neurons present in each tissue.

During molting, both bursicons and ecdysteroids facilitate exocuticle shedding. Ecdysteroids are first secreted as their inactive forms and then converted into their active forms, 20E and ponasterone A (PoA). Ecdysteroid levels correlate with the molting stages in various decapod crustaceans, such as *Callinectes sapidus*, *H. americanus*, *and P. clarkia* (Techa and Chung, 2015). The ecdysteroid level is low in the postmolt stages A-B and the intermolt stage C_0_-C_1_. It gradually increases during the pre-molt stages D_0_-D_2_, peaks at the D_3_ stage, and then sharply declines before ecdysis (Okumura and Aida, 2000). This expression pattern is similar to the *EcR* expression observed in this study (Fig. S1), indicating that the 20E level and expression of *EcR* were correlated. The expression of 20E is related to that of burs hormone, as demonstrated in *Manduca sexta* (Tublitz and Loi, 1993) and *Tribolium castaneum* (Tang et al., 2022). The rising level of burs in the lateral neurosecretory cells is directly triggered by the second 20E surge (Tublitz and Loi, 1993). After ecdysis, the ecdysteroid concentration rapidly decreases, returning to its initial level. This fluctuation in ecdysteroid levels might be linked to the reduced expression of *burs α* and *burs β* observed in the thoracic ganglia of *M. rosenbergii* in this study. Thus, the evidence suggests that the concentration of ecdysteroids in an organism is intricately linked to the expression of *burs α* and *burs β*.

20E binds to the heterodimeric complex of EcR, retinoic acid X receptor (RXR)-orthologue, and a homolog of E75 (Qian et al., 2014). The activated EcR/RXR complex binds to specific hormone response elements (HREs), promoting transcriptional factors, and activates downstream target genes (Abdullah-Zawawi et al., 2021; Lezzi et al., 2002). 20E treatment in *T. castaneum* increases the expression of eclosion hormone and ecdysis triggering hormone and then activates CCAP and burs expression (Tang et al., 2022), cGMP, and other neuropeptides through the transcription factor *SoxC* (Lenaerts et al., 2017; Luo et al., 2022). In *Drosophila,* 20E increases the transcription of peptidoglycan recognition protein-LC (*PGRP-LC*), leading to *antimicrobial peptide* (*AMP*) gene activation through the immune deficiency (IMD) pathway (Rus et al., 2013). This evidence aligns with increased expression of *ALF* and *CHF* in this study, possibly due to this mechanism. Meanwhile, elevated levels of 20E in hemolymph affect *EcR* expression in the regulation of molting crustaceans (Techa and Chung, 2015). Similar to this study, the expression levels of *EcR*, *burs α*, and *burs β* in both thoracic and abdominal ganglia increased following 20E treatment. Thus, burs activate the expression of *AMP* genes through the NF-κB transcription factor, Relish (Li et al., 2019; Liu et al., 2022) like that found in *N. heteropoda* (Li et al., 2019), *P. clarkii* (Zhang et al., 2020), *D. melanogaster* (An et al., 2012), and *A. aegypti* (Zhang et al., 2017).

However, during the molting cycle, the burs hormone and ecdysteroid hormone cause a change in the immune response (Zhang et al., 2021). After molting, the immune system is weakened, making shrimp more susceptible to stress and bacterial infection, and the expression of p38 MAPK decreases (Cheng et al., 2003; He et al., 2013; Liu et al., 2022; Wajsbrot et al., 1990). After treatment with 20E, our work demonstrated that *burs α* and *burs β* were upregulated in the thoracic and abdominal ganglia. This upregulation led to increased expression of *ALF* and *CHF* in hemocytes. However, the exact mechanism by which 20E stimulates *burs* expression requires further study.

**Fig. 8.**
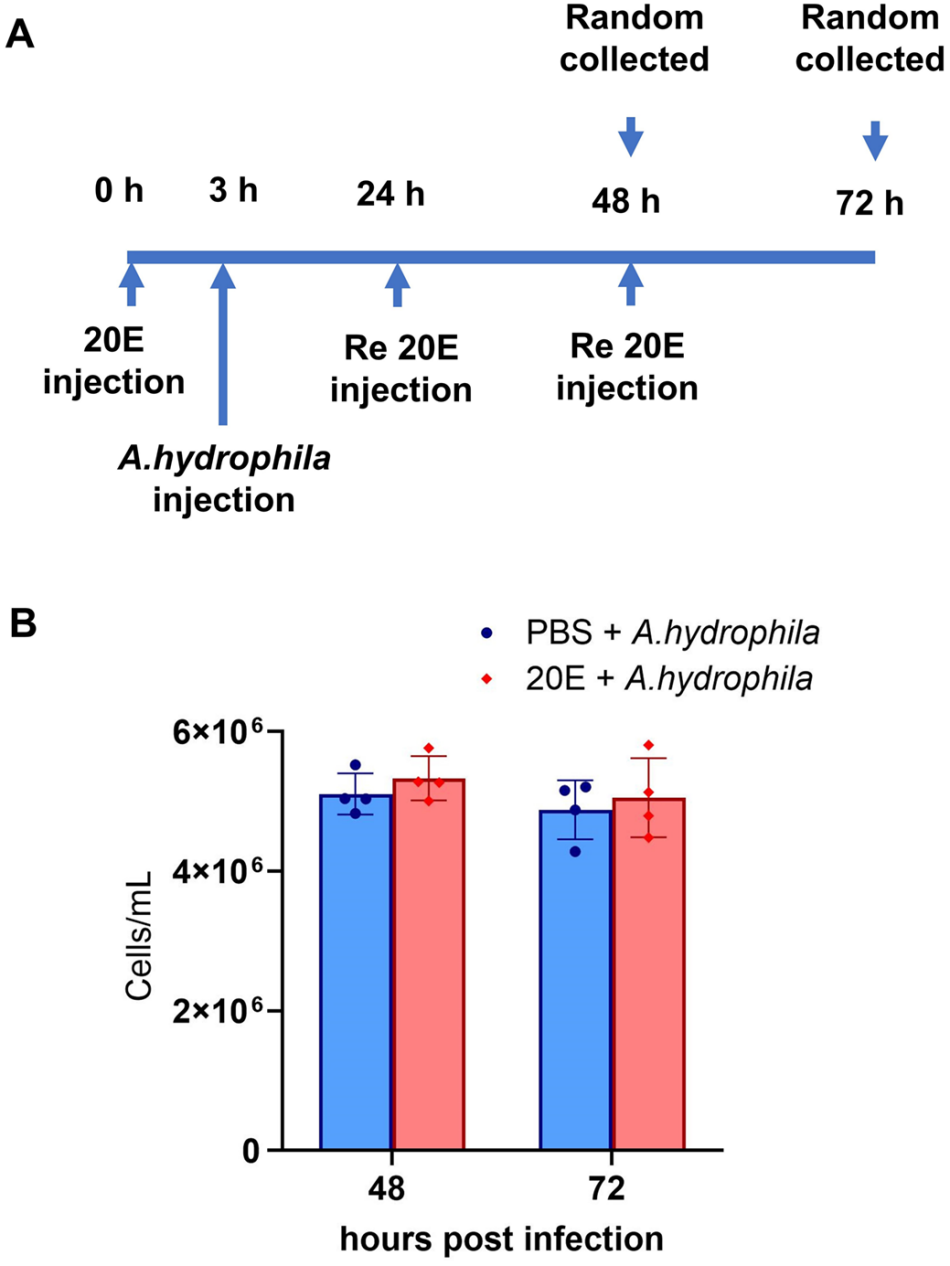
**Retreatment of 20E during *A. hydrophila* infection.** (A) Schematic diagram showing the time point for 20E injection, *A. hydrophila* challenge, and sample collection. The 20E reboost was carried out at 24 and 48 h. (B) Samples collected at 48 h and 72 h show no significant change in the total hemocyte count.

In innate immunity, hemocytes are essential cells produced in hematopoietic tissue and then released into the circulation (Söderhäll and Söderhäll, 2022). Although 20E plays a key role in priming the immune response (Reynolds et al., 2020), it decreases the nonspecific immune response in crustaceans, thus increasing the risk of infection (Wu et al., 2016). In an *in vitro* study, 20E caused a reduction in hemocyte numbers on day 1, which subsequently increased on days 2 and 3 (İzzetoğlu and Karaçalı, 2003). Exposure to 20E may stimulate hemocyte production and circulation within 24 h. This aligns with previous studies, which indicated that 20E regulates hemocyte proliferation and differentiation through hematopoietic division in *D. melanogaster* (Shrivastava et al., 2022) and induces an immune response within 12-18 h in *An. Gambiae* (Reynolds et al., 2020). After 3 h of 20E treatment, hemocyte circulation was significantly increased along with a slight increase in hematopoietic cell proliferation. Additionally, an increase in the number of hemocytes was observed at 6, 12, and 24 h after infection when combined with 20E treatment. This enhances hemocytes' ability to maintain immune function and homeostasis. In *A. gambiae*, 20E has a potential effect on cellular immune function and helps combat microbial infections by boosting the phagocytic activity of hemocytes (Reynolds et al., 2020). However, after 48 h, the hemocyte count returned to normal. When 20E was reinjected every 24 h, it could not stimulate hemocyte circulation (Fig. 8). This effect could be due to the ongoing activity of 20E in combination with an infection, as observed in *A. gambiae* infected with *Plasmodium berghei* (Reynolds et al., 2020). Alternatively, it may be caused by the high concentration of 20E, which is unable to stimulate the production and circulation of hemocytes (Wu et al., 2016). Thus, increased hemocyte circulation within 24 h in response to bacterial infection is an essential process for bacterial clearance and increases the survival rate of prawns (Pudgerd et al., 2021).

Hematopoietic cell proliferation is stimulated by several growth factors and signaling pathways to maintain balance and immune function (Söderhäll and Söderhäll, 2022). In *Bombyx mori*, 20E plays a minor role in stimulating hematopoietic cell proliferation (Nakahara et al., 2003). After 20E treatment, there was an increase in hematopoietic cell proliferation within 24 h, particularly at 12 h. It is possible that 20E activated the expression of *CHF* in hemocytes at 6 and 12 h, as well as in hematopoietic tissue at 6 h. additionally, 20E regulates the expression of several *AMPs* by interacting with peptidoglycan recognition protein (PGRP-LC) (Reynolds et al., 2020; Rus et al., 2013; Sun et al., 2016), PGRP-SA (Han et al., 2017) or directly enhancing enhances Broad-Complex Z2 (Br-C Z2) (Ma et al., 2019). In this study, we observed upregulation of both *burs α* and *burs β* in the thoracic and abdominal ganglia after 20E treatment. There is evidence indicating that the formation of a burs homodimer results in upregulation of *AMPs*, including *crustin*, *carcinin*, *lysozyme*, and *ALF* (Liu et al., 2022; Zhang et al., 2020). Therefore, 20E may serve two functions: (1) directly activating *AMPs* and (2) indirectly activating *AMPs* through the expression of *burs*. In this study, we demonstrated upregulation of *ALF* at 6 h in hemocytes and hematopoietic tissue at 6 and 12 h after 20E treatment during *A. hydrophila* infection. ALF is an antimicrobial agent that binds to the cell wall of gram-negative bacteria to neutralize lipopolysaccharides (Cerenius et al., 2008). Meanwhile, *CHF* expression prevents apoptosis of HPT cells and hemocytes (Li et al., 2021). These genes are highly expressed in both hematopoietic tissue and hemocytes during bacterial infection in *M. rosenbergii* (Pudgerd et al., 2021). However, this study has limitations, as we did not use inhibitors or gene silencing techniques to directly test the effects of 20E and bursicon on immunity. From previous studies, chemical inhibitors, ucurbitacins, or *EcR* gene silencing have been explored in *B. mori* and *D. melanogaster* larvae, having a potential effect on growth by blocking molting and metamorphosis, respectively, as well as inhibiting vitellogenin expression in *Scylla paramamosain* induced by 20E signaling (Zou et al., 2018; Gong et al., 2015). Thus, in this case, inhibition of 20E signaling could cause alteration of burs expression and immune modulation.

The prophenol oxidase system is a component of humoral immunity and an important mechanism for resisting pathogen infection. The proPO activating enzyme is initially inactive and becomes active PO through catalysis and proteolysis, resulting in the melanization of the pathogen (Liu et al., 2006; Pudgerd et al., 2021). In American cockroaches, the activity of blood phenol oxidase is highest during the molting process and slightly less active at other times (Mills et al., 1968). 20E enhances the proPO signaling cascade through gram-negative binding proteins-2 (GNBP-2) in *L. migratoria* (Sun et al., 2016). Similar to our study, 20E enhanced the upregulation of *proPO* in hemocytes and increased PO activity in the hemolymph of bacterial-infected prawns within the first 24 h. Additionally, the burs hormone regulates cuticular tanning, a process that requires expression of the PO gene (Song and An, 2011). Thus, the phenol oxidase system might be activated by burs hormone (Mills et al., 1968). This study demonstrated that following treatment with 20E and infection with *A. hydrophila*, there was a significant increase in the expression of *burs* within the first 12 h, together with elevated expression of *proPO* in hemocytes and increased PO activity in the hemolymph within 24 h. However, further investigation is needed to understand the relationship between *burs* expression and PO activity. Additionally, 20E treatment led to increased expression of *burs* and immune-related genes, resulting in enhanced survival of *M. rosenbergii* following infection with *A. hydrophila*. The results aligned with prior studies, where 20E treatment activated the immune response (Rus et al., 2013; Reynolds et al., 2020; Sun et al., 2016; Wang et al., 2022), enhanced the survival rate of silkworms infected with *Escherichia coli* and *Bacillus bombyseptieus* (Sun et al., 2016), and *L. migratoria* infected with *Metarhizium anisopliae* (Han et al., 2017).

In conclusion, this study showed that the ecdysteroid hormone plays a dual role in enhancing the immune system of *M. rosenbergii*. Treatment of infected prawns with 20E increased the survival rate. This was accompanied by activation of *burs α* and *burs β* gene expression, which in turn promoted immune-related gene expression and increased hemocyte production. Additionally, 20E directly activated the expression of immune-related genes. These findings improve our understanding of the molecular crosstalk mechanism between ecdysteroid hormone and burs in regulating the immune system. The results of this study provide valuable information on the regulation of innate immunity during the molting period. This could potentially lead to the development of hormonal supplements that may help protect prawns from disease, offering a new disease prevention strategy in freshwater aquaculture. However, 20E injections may render the prawns more susceptible to infection after the 20E-induced molt, and the molecular mechanism of ecdysteroid-induced burs hormone activation remains to be investigated.

## MATERIALS AND METHODS

### Animal experiments

*M. rosenbergii* (20-30 g in body weight) was obtained from a local farm in Suphanburi Province, Thailand. They were screened for specific pathogens, WSSV, *Mr*NV, and DIV1, by PCR and RT-PCR. The prawns were stocked in tanks (500 l), aerated under a natural photoperiod condition and fed daily with commercial pellets. All animal studies were performed with the approval of the Laboratory Animals Research Center of the University of Phayao and approved by the Animal Ethics Committee of the University of Phayao (approval number: 1-010-66).

Healthy prawns in the molt stage were selected by observing the development of the pleopods' setae under a light microscope and the exoskeleton texture based on previously reported criteria (Kamaruding, et al., 2018). The molting cycle consists of three main stages: post-molt, intermolt, and pre-molt. Additionally, each stage consists of sub-stages: post-molt (sub-stages A and B), intermolt (sub-stages C_0_ and C_1_), and pre-molt (sub-stages D_0_, D_1_, D_2_, and D_3_).

Prawns were ice-cold anesthetized, and hemolymph (100 µl) was collected by withdrawal from the pericardial sinus of each prawn into a 1 ml sterile syringe (23 gauge) containing 900 µl of Alsever's anticoagulant solution (Cheng et al., 2003). For hemocyte enumeration, the anticoagulant-hemolymph mixture was placed on a hemocytometer to determine the total hemocyte count (THC) using a light microscope (Leica ICC 50HD). Differential hemocyte counting (DHC) was performed by Bengal Rose staining (Sritunyalucksana et al., 2005). The hematopoietic tissue and the thoracic and abdominal ganglia were collected for expression analysis of the immune-related genes *burs α* and *burs β*, respectively.

### 20E injection and *A. hydrophila* challenging

To investigate whether 20E affects the immune response of *M. rosenbergii*, a stock of 10 mg/ml 20E (20-hydroxyecdysone, Sigma) was dissolved in ethanol and diluted with phosphate buffered saline (PBS) to a final concentration of 100 µg/ml for injection. The group of five prawns (stage C_0_) was given an intracardiac injection with 20E (10 ng/g prawn), and a control group was injected with an equal volume of PBS. Three hours post-injection, the hemolymph, hematopoietic tissue, and thoracic and abdominal ganglia were collected for investigation of *burs* expression and immune status. For gene expression analysis, three individual prawns were pooled to form one biological replicate, resulting in *n*=4-5 replicates per group per time point.

To determine the effect of 20E on immune potency against *A. hydrophila* infection, a group of prawns was given 10 ng/g prawn of 20E by intracardiac injection. After 3 h, these groups of prawns were intramuscularly injected with 100 µl of PBS containing LD_50_ of *A. hydrophila* (8.91×10^5^ CFU/ml). The details of the bacterial culture, method, and calculation of LD_50_ are shown in Supplementary Data 1 and Fig. S2. The prawns from each group were randomly collected at 6, 12, 24, and 48 h post-infection. The hemolymph, hematopoietic tissue, thoracic ganglia, and abdominal ganglia were removed and processed for analysis. The mortality rate of prawns challenged with *A. hydrophila* and injected with 20E was compared with that of a control group receiving PBS treatment. The mortality rate experiment was carried out with three replicates.

### Mitotic hematopoietic cell counting

The hematopoietic tissue was removed and preserved in Davison's fixative for 24 h. Subsequently, it was washed with 70% ethanol. The tissue was then processed for paraffin embedding and cut into 5 µm sections. Following the standard protocol, the tissue section was stained with hematoxylin (Merk KGaA, Germany) and eosin (Merk KGaA, Germany) solution. Images were captured under a microscope (Nikon Upright Microscope Eclipse Ni-U) at 40× magnification. For quantitative analysis, the hematopoietic cells that underwent cell division, as indicated by the characteristics of the nucleus, were manually counted in four areas within a 40× field.

### Phenoloxidase activity analysis

A 100 µl volume of hemolymph was withdrawn from the heart of the prawn into a 1 ml syringe containing 900 µl of TSB-I (Jariyapong et al., 2020). The mixed hemolymph was transferred to 1.5 ml centrifuge tube and subsequently centrifuged at 12,000× ***g*** to separate hemocyte for 15 min at 4°C. Then, the hemolymph was stored at −80°C for further analysis of PO activity. For hemocyte collection, 500 µl of hemolymph was withdrawn from the heart into a 1 ml syringe containing 500 µl of Alsever's solution. The mixed hemolymph was centrifuged at 12,000× ***g*** for 15 min at 4°C to obtain the hemocyte pellet. The supernatant was discarded, and the hemocyte pellet was washed with cacodylate-citrate buffer (Cheng and Chen, 2000). After centrifuging and discarding the supernatant, the hemocyte pellet was then resuspended with 200 µl of cacodylate buffer (Cheng and Chen, 2000) and stored at −80°C for further analysis. PO activity in the hemolymph and hemocyte was measured by recording the formation of dopachrome production from L-dihydroxyphenylalanine (L-DOPA) following the procedures outlined in previous studies (Cheng and Chen, 2000; Jariyapong et al., 2020).

### Quantitative real-time PCR (qPCR)

Total RNA from the various tissues of *M. rosenbergii* was isolated using Tri Reagent^®^ (Molecular Research Center, Inc.), according to the manufacturer's protocol. The quality and quantity of RNA were assessed by measuring their absorbance at 260 and 280 nm using NanoDrop One (Thermo Fisher Scientific). One microgram of RNA from each sample was treated with DNase I to eliminate DNA contamination. The RNA was then subjected to cDNA synthesis using Random Hexamers (Invitrogen, USA) and SuperScript^®^ III Reverse Transcriptase (Invitrogen, USA), following the manufacturer's protocol. The completed cDNA solution was stored at −20°C for further analysis.

The relative expression of the genes (*Burs α*, *Burs β*, *ALF*, *CHF*, and *EcR*) in the thoracic ganglion, abdominal ganglion, hematopoietic tissue, and hemocytes was measured using qPCR with a QIAquant 96, 5plex (Qiagen). Amplification was performed using a 96-well plate in a 20 µl reaction volume. Each reaction contained 1 µl of cDNA from each tissue, 10 µl of SensiFAST^TM^ SYBR^®^ No-ROX kit (Bioline), 0.4 µl of each primer (10 µM/µl), and 8.2 µl of sterile dH_2_O. In the thermal qPCR cycle profile, the following steps were followed based on the manufacturer's protocol: an initial cycle of 95°C for 2 min for enzyme activation, followed by 40 cycles of 95°C for 5 s for denaturation, and 60°C for 20 s for annealing and extension. Sterile distilled water (dH_2_O) was used as the negative control instead of the template. The same qPCR cycle profile was applied to the internal control gene *EF-1α*. Details of the primers used in this experiment are shown in Table 1. After completing the qPCR program, the data were analyzed using QIAquant96 software, with the baseline set automatically to ensure consistency. The comparative CT method (2^−ΔΔCT^ method) was used to analyze the expression level of immune-related genes (Livak and Schmittgen, 2001). The results were expressed as the mean±s.d. of the relative fold change for one sample.

**Table 1.** Specific primers used in this experiment

Primer	Sequences	Amplicon size	References
*Mr*Bur-α-F	5′-TTCACTTGGACCCTGATGCC-3′	325 bp	Jariyapong et al., 2020
*Mr*Bur-α-R	5′-CCTCGATGTCAGTGCAAGGT-3′		
*Mr*Bur-β-F	5′-TGGCAATGAACCCCTGTACC-3′	357 bp	Jariyapong et al., 2020
*Mr*Bur-β-R	5′-CGCATTTAGCACACTGGCAA-3′		
*Mr*ALF-F	5′-GTCTTGGGTTGTTTTGGTAA-3′	254 bp	Jariyapong et al., 2019
*Mr*ALF-R	5′-CATCGTTACTTCCCACTTGT-3′		
*Mr*CHF -F	5′-GAGGGTCTGTCTTGCTACTG-3′	220 bp	Jariyapong et al., 2019
*Mr*CHF -R	5′-GGTACTTCTCCTCGTCTCCT-3′		
*Mr*proPO -F *Mr*proPO -R	5′-ACACTGAAGGACATAAGGCGAGAT-3′ 5′-AGTAGAGTTCCAAGTCGGAGATGCT-3′	70 bp	Liu et al., 2006; Thansa et al., 2021
*Mr*EF1α-F	5′-ATGTCATGGTGGAAGAAGAG-3′	219 bp	Jariyapong et al., 2019
*Mr*EF1α-R	5′-AAAGTTGACCACCATACCAG-3′		
*Mr*EcR-F	5′-TTGAGATCAAAGTCGCCCCC-3′	210 bp	KM886340.1
*Mr*EcR-R	5′-GAGCTCGACACTTCCGAACA-3′		

### Statistical analysis

The data are presented as the mean±s.d.. One-way ANOVA and Tukey's multiple comparison test were performed to compare the differences between the molting stages. Statistical significance between the two groups was analyzed using an unpaired *t*-test, with the significance level set at *P*<0.05. All quantified data were plotted and analyzed using GraphPad Prism 9.0.0 software.

## Supplementary Material

10.1242/biolopen.061773_sup1 Supplementary information
