# Supplementary material for: Inducing bursicon expression using 20-hydroxyecdysone (20E) increased immune response in Macrobrachium rosenbergii against Aeromonas hydrophila
Source: Biol Open. 2025 Jul 16;14(7):bio061773. doi: 10.1242/bio.061773 (PMC12309897; doi:10.1242/bio.061773)
Supplement: Supplementary information [file biolopen-14-061773-s1.pdf]

Supplementary Data 1.

***A. hydrophila* culture**

A. hydrophila was cultured overnight with tryptic soy broth (TSB) at 30 °C with continuous shaking until OD_{600} reached 0.6. The bacterial cells were collected by centrifugation at 8,500 × *g* for 10 min at 4°C and washed with PBS three times. The bacterial pellet was resuspended in 0.1M PBS (pH 7.4). Ten-fold serial dilution was performed, starting from 1:10 to 1:100,000. One hundred microliters of each dilution were spread on tryptic soy agar (TSA) and incubated for 16 h at 30 °C. The number of bacterial colonies was calculated as colony-forming units per milliliter (cfu/mL).

Bioassay and experimental challenge for calculating LD_{50}

The bacteria were washed with 1X PBS and resuspended in 1X PBS. The bacterial solution was prepared at the concentrations of 1×10^3–1×10^9 cfu/mL for the challenging experiment. The prawn was divided into 8 groups (n = 15 per group). The control group, prawns were intramuscularly injected with 100 µl, whereas the experimental groups were intramuscularly injected with 100 µL of PBS containing *A. hydrophila*. The prawns were maintained for 5 days and the mortality was observed every 12 h. This experiment was performed in triplicate. LD_{50} value was determined and calculated as cfu/mL by Probit analysis. The calculation of LD_{50} value was 8.91×10^5 CFU/ml

Biology Open • Supplementary information

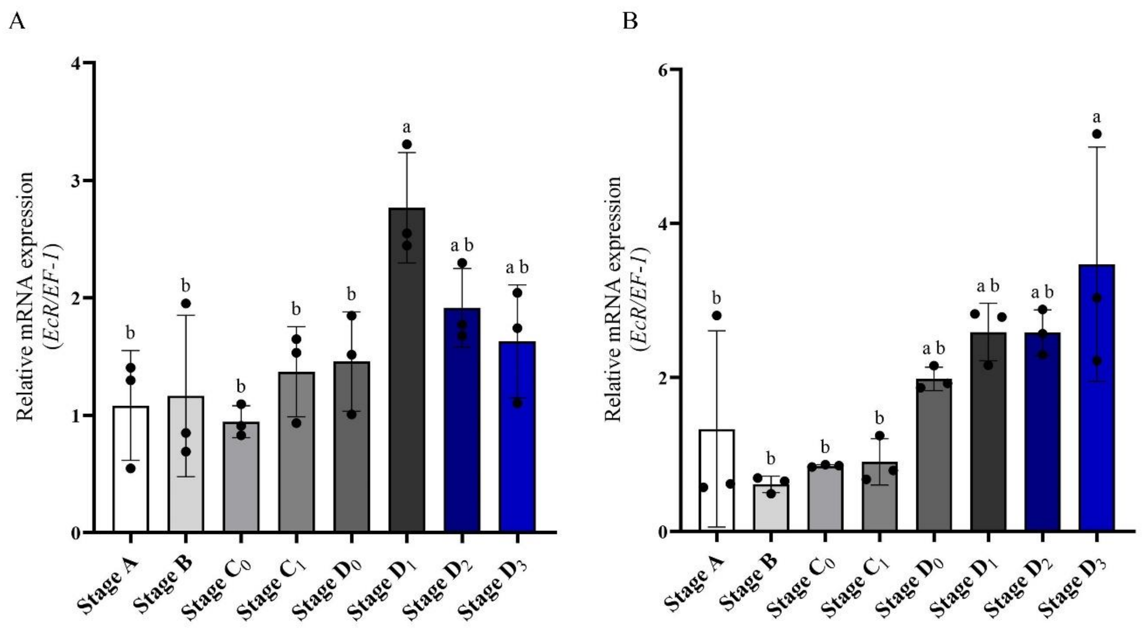

Fig. S1. *EcR* expression during the molt stage. The mRNA expression of *EcR* in the thoracic ganglion (A) and abdominal ganglion (B) (n=3). Different letters indicate significant differences between the molt stages ($p < 0.05$).

Biology Open • Supplementary information

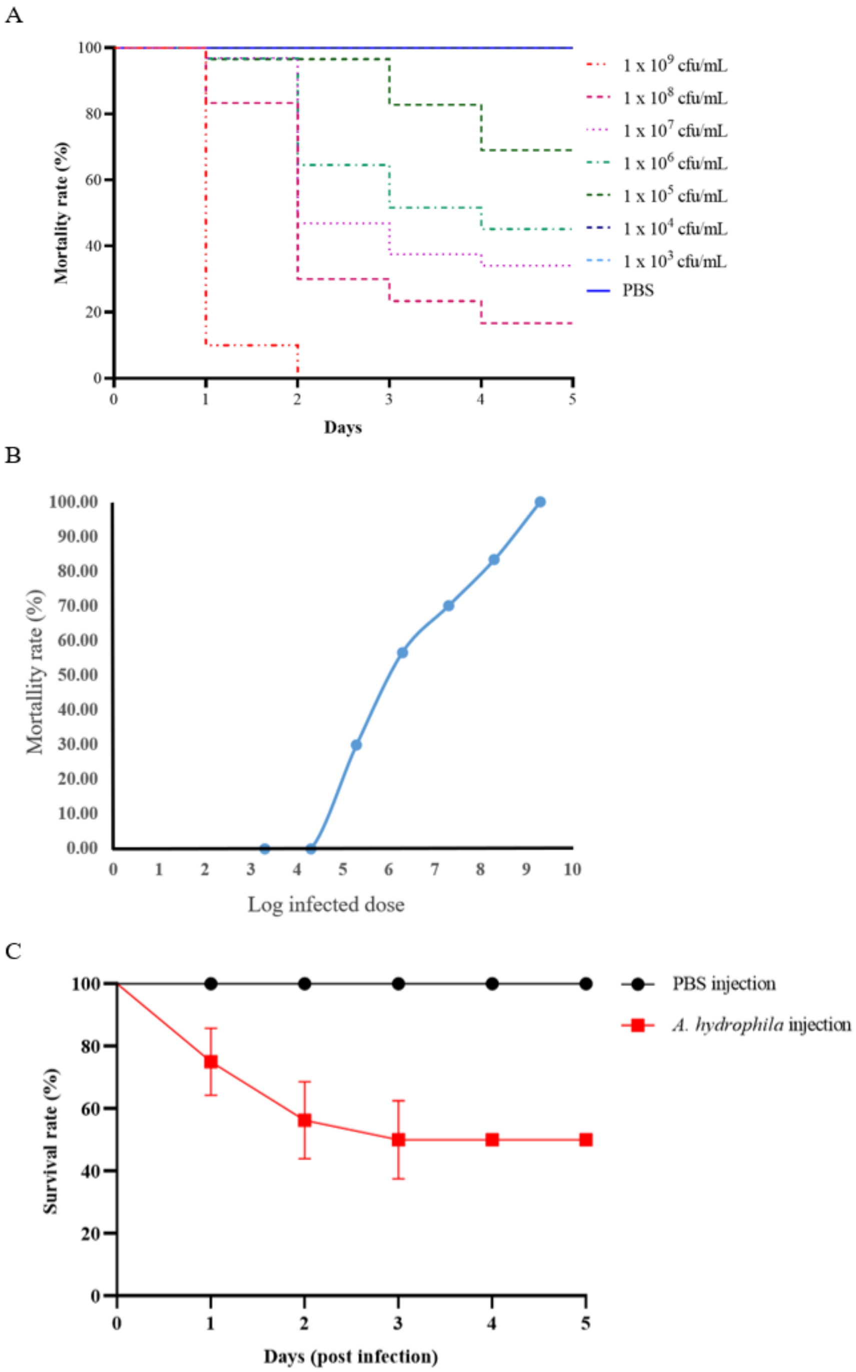

Fig. S2. Survival rate of *M. rosenbergii* challenged with *A. Hydrophila*. To determine the LD_{50} dose of *A. hydrophila*. The *M. rosenbergii* was intramuscularly injected with various concentrations of *A. hydrophilla*. The mortality of the prawn was observed to be related in a dose-dependent manner (A). No mortality was observed in *M. rosenbergii* infected with 1 × 10^3 and 1 x 10^4 cfu/mL as well as in controls. (B) The graph showed a plot of *M. rosenbergii* mortality after *A. hydrophila* infection. (C) The survival rate of *M. rosenbergii* challenged with LD_{50} doses of *A. hydrophila* was analyzed with the Kaplan-Meier method by GraphPad Prism 9.0.0.

Biology Open • Supplementary information
